# A convenient method for the accurate identification of Citri Reticulatae Pericarpium using image and multi-stream

**DOI:** 10.1371/journal.pone.0340161

**Published:** 2026-02-05

**Authors:** Zhiyi Wu, Tianshu Wang, Zhongyuan Mao, Lizhi Huang, Jiyuan Chen, Xichen Yang

**Affiliations:** 1 The School of Artificial Intelligence and Information Technology, Nanjing University of Chinese Medicine, Nanjing, China; 2 The School of Computer and Electronic Information/School of Artificial Intelligence, Nanjing Normal University, Nanjing, China; Guizhou University, CHINA

## Abstract

*Citri Reticulatae Pericarpium* (CRP), the dried peel of citrus fruits, holds notable dietary and medicinal value. Its quality and price largely depend on origin and aging. Lower-grade CRP is often adulterated to imitate premium products, making accurate authentication of region and vintage essential for quality assurance and fair market valuation. Existing methods for vintage classification are limited due to complex equipment and high operational costs, restricting their scalability in practical applications. To address these issues, a convenient method for the accurate identification of Citri Reticulatae Pericarpium using image and multi-stream is proposed. The method comprises three main stages. Firstly, an object detection network with bounding box refinement localizes exocarp and albedo regions from whole CRP images. Secondly, a three-stream feature extractor processes the whole images along with exocarp and albedo patches to capture complementary visual details. A channel-level feature interaction module further enhances robustness through cross-region feature integration. Thirdly, a meta-learning module enables rapid adaptation to images captured under varying conditions by different consumer-grade devices. Experimental results demonstrate that the proposed method achieves an accuracy of 95.5% on iPhone-captured images. In addition, for images captured by different devices, the proposed method achieves a relative accuracy improvement of more than 34% over the direct transfer method, mainly owing to the meta-learning adaptation to different devices.

## Introduction

*Citri Reticulatae Pericarpium* (CRP), commonly known as citrus peel, is a major by-product of the global citrus industry [1]. CRP has a distinctive flavor that enhances the palatability of food. In addition, it exhibits significant effects on improving digestion and energy metabolism [2]. As a dual-purpose edible and medicinal substance, CRP demonstrates versatile applications [3,4]. It can be processed into herbal tea through hot water extraction, or manufactured into leisure foods such as multi-processed CRP and CRP-prune snacks.. In culinary applications, CRP is commonly utilized as a natural seasoning added to braised meats and curries to reduce greasiness and eliminate odors, while also being incorporated into desserts to enhance flavor This diversified use reflects both traditional dietary wisdom and broad applicability in the modern food industry.

The quality of CRP is determined not only by its geographical origin but also significantly influenced by its aging duration. The CRP from the Xinhui region of Guangdong is considered the highest quality for its superior pharmacological effects and its rich cultural heritage [5]. Moreover, the medicinal and market value of CRP increases significantly with extended aging duration. It has been scientifically confirmed that as CRP ages, beneficial flavonoids accumulate progressively, while distinctive aroma compounds undergo gradual formation [6,7]. Thus, Xinhui CRP with longer aging periods is significantly more expensive than newly harvested CRP from other regions. However, lower-grade CRP is often adulterated by simulating aged appearance and then fraudulently marketed as a premium product [8,9]. Common consumers face significant challenges in differentiating premium-grade CRP from adulterated or lower-quality products. Therefore, a reliable, scalable, and cost-effective authentication method is urgently needed for CRP vintage and origin classification.

Current methods for classifying the vintage and origin of CRP mainly rely on analytical techniques, including near-infrared spectroscopy [10–12], hyperspectral imaging [13], Raman spectroscopy [14], and terahertz spectroscopy [15]. Metabolomics is also used to analyze chemical fingerprints for differentiation [16–18]. To interpret the resulting high-dimensional data, conventional machine learning methods are commonly applied [19,20]. While effective under controlled conditions, these approaches often fail to maintain accuracy in real-world scenarios, as they depend on expensive instruments, complex procedures, and incur high testing costs. These limitations hinder scalability and compromise the applicability of existing methods for rapid, low-cost classification in commercial settings, prompting growing interest in computer vision-based deep learning methods as an alternative.

Deep learning is an effective approach for food-related plant classification, building upon significant advances in image recognition from AlexNet [21] through modern convolutional neural network (CNN) architectures [22–25]. It enables precise detection of defects, grading, and species identification in agricultural and food products by extracting discriminative features from complex visual scenes, thereby enhancing both efficiency and accuracy [26–28]. Despite this progress, research on commercially valuable food products with dual dietary and medicinal significance, such as CRP, remains limited. Existing studies, including a lightweight model based on the Cross Stage Partial Network (CSPNet) proposed by Chu et.al [29] and the ConvNeXt approach with attention mechanisms developed by Deng et.al [30], predominantly employ single-input frameworks. These models emphasize global features while overlooking fine-grained, multi-stream visual cues present in both the exocarp and albedo layers of CRP. In addition, Zhang et al. [31] developed a non-destructive, data-driven method for CRP aging assessment, further highlighting the importance of data-driven approaches for CRP quality evaluation. Food materials like CRP often exhibit complex, hierarchically structured visual traits under diverse imaging conditions. Multi-stream deep feature fusion methods, which integrate global and local features, have shown effectiveness in food quality inspection [32–35]. Therefore, applying a multi-stream deep feature fusion strategy is expected to markedly enhance CRP vintage classification by enabling precise extraction of its intricate morphological characteristics.

In real-world scenarios, images captured by consumer devices vary significantly in resolution, lighting, and perspective, causing domain shift—a major obstacle for deep learning generalization [36]. Meta-learning, particularly Model-Agnostic Meta-Learning (MAML) [37], addresses this by enabling rapid adaptation to new data distributions from limited samples, thus significantly enhancing cross-domain generalization [38,39]. Recent studies have demonstrated meta-learning’s effectiveness across various domains, including fine-grained image recognition [40], hyperspectral classification [41,42], medical imaging diagnosis [43,44], low-data scenarios [45,46], natural language understanding [47], and plant classification under environmental variability [48]. These works collectively confirm the robust ability of meta-learning to mitigate domain shifts and improve generalization in diverse applications.

To address the issues discussed above, a convenient method for the accurate identification of Citri Reticulatae Pericarpium using image and multi-stream is proposed. First, an object detection network with bounding box refinement is used to localize exocarp and albedo regions in CRP images. A three-branch network with multi-stream feature fusion and feature interaction is then used to process the whole images, exocarp, and albedo patches to capture complexity visual information. A meta-learning module is finally applied to enable rapid adaptation to images captured by different devices. Extensive experiments demonstrate the method’s superior accuracy and robustness across imaging conditions.

Our contributions are summarized as follows:

1. Proposing a consumer-grade classification framework for CRP based on images. An object detection network with a bounding box refinement algorithm extracts exocarp and albedo patches from whole CRP images. A three-branch multi-stream feature fusion network is then designed to extract global and local features from the whole image, exocarp, and albedo patches.
2. A meta-learning module enables rapid adaptation across images from various mobile devices. This allows the proposed method to generalize across varying imaging conditions caused by hardware differences and achieve accurate classification without the need for specialized instruments.
3. Experimental results demonstrate that the proposed method achieves an accuracy of 95.5% on the iPhone-captured dataset and also exhibiting strong cross-domain generalization.

## Materials and methods

### Image acquisition

**Fig 1:**
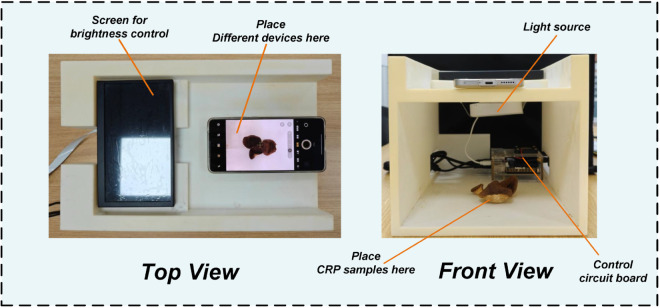
Image acquisition device with top and front views.

To ensure consistency in lighting and positioning, a custom image acquisition device was constructed, as illustrated in Fig 1. The device features an LCD screen to control ambient brightness, integrated LED light sources, and a control circuit board. Smartphones were placed at the marked position to capture images under standardized conditions. Both top and front views of the setup are provided to illustrate its structure.

To support vintage classification and cross-device generalization analysis, a dataset comprising 399 CRP specimens with varied price points is constructed, encompassing differences in origin, aging duration, and authenticity. Specifically, the dataset includes: Counterfeit CRP samples were collected from Wuzhou, Guangxi Province , priced at 190 CNY per kilogram with a total of 120 slices and Yunfu, Guangdong Province, priced at 560 CNY per kilogram with a total of 105 slices, both marketed as premium-grade products originating from Xinhui. Genuine CRP samples were sourced from Xinhui, Guangdong Province, including those aged over 10 years, priced at 2800 CNY per kilogram with a total of 84 slices and over 15 years, priced at 3300 CNY per kilogram with a total of 90 slices. The labels are assigned based on price categories. Detailed information for each class is summarized in Table 1.

**Table 1:** Detailed information of the CRP dataset.

Label	Place of origin	Specification	Unit Price (CNY/kg)	Sample Count
190	Wuzhou, Guangxi, China	Counterfeit	190	120
560	Yunfu, Guangdong, China	Counterfeit	560	105
2800	Xinhui, Guangdong, China	Over 10 years	2800	84
3300	Xinhui, Guangdong, China	Over 15 years	3300	90

In the dataset, all CRP samples were photographed using three mobile devices of different brands and price levels. This introduced domain shifts due to device-specific variations. Detailed information on the devices is provided in Table 2.

**Table 2:** Camera specifications of acquisition devices.

Device	Main Sensor	Specification
iPhone 14 Pro Max	Sony IMX803	48 MP, 1/1.28", 1.22 μm (original)/2.44 μm (binning), Stacked BSI CMOS, Quad-Bayer, OIS Support
Xiaomi Pad 6 Pro	Samsung S5KJN1	50 MP, 1/2.76", 0.64 μm (original)/1.28 μm (binning), ISOCELL 2.0, Tetrapixel
Vivo X80	Sony IMX866	50 MP, 1/1.56", 1.0 μm, Stacked CMOS, RGBW Array, PDAF, OIS Support, Multi-Aspect Ratio Output

Meanwhile, to enrich the visual information of CRP specimens, this study captured images of both the front exocarp and the back albedo. The front exocarp images reveal surface texture and overall morphology, while the back albedo images expose color, texture, and fibrous structures that provide crucial cues for assessing vintage and authenticity.

**Fig 2:**
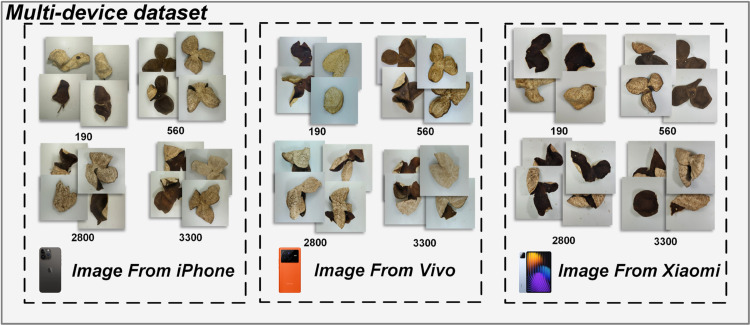
Visual differences in CRP images captured by different devices.

Fig 2 shows CRP images captured by iPhone, Vivo, and Xiaomi devices. Differences in hardware and processing lead to noticeable shifts in color, sharpness, and brightness, causing domain gaps that hinder model generalization.

### Method

**Fig 3:**
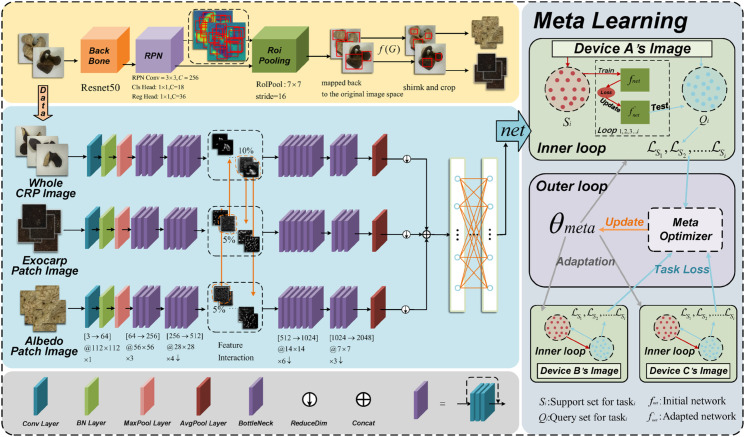
Overall architecture of the proposed method.

In this paper, a consumer-grade CRP vintage classification method via multi-stream deep feature fusion and meta-learning is proposed. As illustrated in Fig 3, the method comprises three main modules. First, an object detection network with bounding box refinement accurately localizes and extracts the exocarp and albedo patches from the whole CRP images. Then, multi-stream feature extraction is performed by separately feeding the whole image, the exocarp patch, and the albedo patch into three branches of deep networks. A cross-channel interaction mechanism further enhances information exchange among these branches, improving feature representation. Finally, meta-learning optimization constructs cross-device training tasks, enabling rapid adaptation to diverse imaging devices and boosting generalization under heterogeneous environments.

#### Object detection.

In this method, object detection and bounding box refinement are employed to accurately localize key regions of CRP and provide high-quality inputs for subsequent feature extraction. The process consists of the following three steps.

First, a Faster Region-based CNN (Faster R-CNN) object detection network is adopted to automatically generate candidate regions and predict both object categories and bounding box coordinates. Its strong balance between detection accuracy and computational efficiency makes it well-suited for precise localization tasks in limited-data scenarios. The object detection process can be formulated as:

$$𝐁=D_{FRCNN}(I),$$ (1)

where $D_{FRCNN}(\cdot )$ denotes the Faster R-CNN detection process, and $𝐁=\{b_{1},b_{2},\ldots ,b_{n}\}$ is the set of detected bounding boxes.

Then, the global grayscale mean *μ* of the image *I* is computed:

$$\mu =\frac{1}{H\times W}\sum_{i=1}^{H}\sum_{j=1}^{W}I(i,j),$$ (2)

where *H* and *W* denote the height and width of the image, and *I*(*i*,*j*) is the grayscale value at pixel (*i*,*j*).

For each bounding box $b=(x_{1},y_{1},x_{2},y_{2})$, the grayscale values at the four corners are extracted as:

$$\{I_{TL}=I(x_{1},y_{1}),\;I_{TR}=I(x_{2},y_{1}),\;I_{BL}=I(x_{1},y_{2}),\;I_{BR}=I(x_{2},y_{2}),$$ (3)

where the subscripts TL, TR, BL, and BR denote the top-left, top-right, bottom-left, and bottom-right corners of the bounding box, respectively. The absolute deviations of each corner value from the mean are computed as:

$$\{\Delta_{TL}=|I_{TL}-\mu |,\;\Delta_{TR}=|I_{TR}-\mu |,\;\Delta_{BL}=|I_{BL}-\mu |,\;\Delta_{BR}=|I_{BR}-\mu |.$$ (4)

The deviation threshold *τ* used in Algorithm 1 is determined from the average gray levels of the CRP region and the background. Let $\mu_{\text{CRP}}$ and $\mu_{\text{bg}}$ denote the mean gray values of the CRP region and the background, respectively. We first compute their gray-level difference:

$$d=\left|\mu_{\text{CRP}}-\mu_{\text{bg}}\right|.$$ (5)

The deviation threshold is then set to half of this difference:

$$\tau =\frac{d}{2}=\frac{\left|\mu_{\text{CRP}}-\mu_{\text{bg}}\right|}{2}.$$ (6)

According to tests on our dataset, the gray-level difference between $\mu_{\text{CRP}}$ and $\mu_{\text{bg}}$ is around 40. Therefore, we set τ=20 and use it as the deviation threshold in the subsequent bounding-box refinement.

Using the global mean *μ* and the threshold *τ*, dynamic bounding box refinement is applied by iteratively adjusting the box inward if the deviation at any corner exceeds *τ*, proceeding with a fixed step size until all deviations fall below the threshold. This strategy ensures that each bounding box tightly encloses the target region, improving the accuracy and robustness of subsequent feature extraction. The detailed procedure is presented in Algorithm 1.

**Algorithm 1 Bounding box refinement based on grayscale deviation.**

**Require:** Bounding box $b=(x_{1},y_{1},x_{2},y_{2})$; shrink step *s*; threshold *τ*

**Ensure:** Refined bounding box $\left(x_{1},y_{1},x_{2},y_{2}\right)$

1: Compute image grayscale mean *μ*

2: **repeat**

3:   $I_{TL}\leftarrow I(x_{1},y_{1})$;   $I_{TR}\leftarrow I(x_{2},y_{1})$

4:   $I_{BL}\leftarrow I(x_{1},y_{2})$;   $I_{BR}\leftarrow I(x_{2},y_{2})$

5:   $\Delta_{TL}\leftarrow |I_{TL}-\mu |$;   $\Delta_{TR}\leftarrow |I_{TR}-\mu |$

6:   $\Delta_{BL}\leftarrow |I_{BL}-\mu |$;   $\Delta_{BR}\leftarrow |I_{BR}-\mu |$

7:   **if** $\Delta_{TL}>\tau$ **then**

8:    $x_{1}\leftarrow x_{1}+s$;   $y_{1}\leftarrow y_{1}+s$

9:   **end if**

10:   **if** $\Delta_{TR}>\tau$ **then**

11:    $x_{2}\leftarrow x_{2}-s$;   $y_{1}\leftarrow y_{1}+s$

12:   **end if**

13:   **if** $\Delta_{BL}>\tau$ **then**

14:    $x_{1}\leftarrow x_{1}+s$;   $y_{2}\leftarrow y_{2}-s$

15:   **end if**

16:   **if** $\Delta_{BR}>\tau$ **then**

17:    $x_{2}\leftarrow x_{2}-s$;   $y_{2}\leftarrow y_{2}-s$

18:   **end if**

19: **until** All *Δ* values <τ

20: **return** $\left(x_{1},y_{1},x_{2},y_{2}\right)$

**Fig 4:**
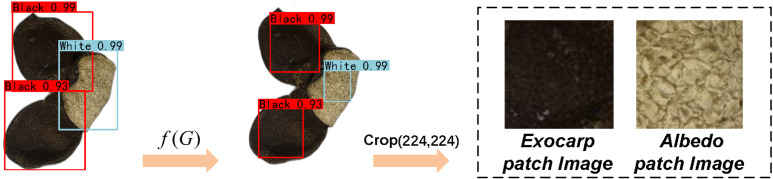
Illustration of object detection and bounding box refinement.

Fig 4 illustrates the overall object detection process, where f(G) denotes the proposed bounding box refinement algorithm.

#### Multi-stream feature extraction.

**Fig 5:**
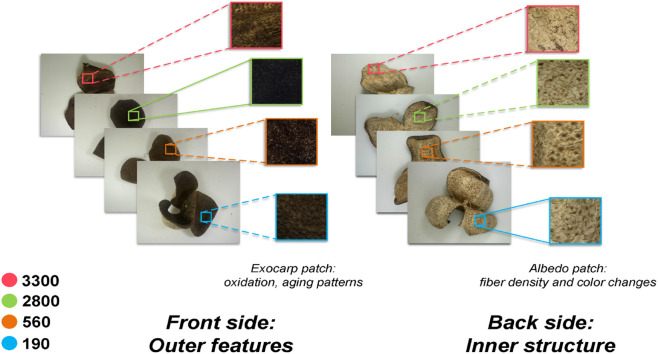
CRP images with extracted key regions, illustrating the exocarp patch and albedo patch used for vintage-related feature analysis.

A three-branch multi-stream feature extraction framework based on ResNet50 is employed. Specifically, three parallel extraction branches are designed for the whole CRP image, the exocarp patch and the albedo patch.An example of the extracted exocarp and albedo patches from a CRP sample is shown in Fig 5. This design enables the model to effectively characterize the overall morphology, the surface texture of the outer skin, and the internal tissue architecture, respectively. Features extracted from the whole image emphasize global texture and contour morphology, facilitating macroscopic discrimination. The exocarp patch focuses on capturing fine-grained surface texture details, revealing subtle microstructural variations. Meanwhile, the albedo patch encodes the structural state of the internal capsule tissue. The integration of these three complementary feature scales provides a holistic representation of CRP’s distinctive characteristics across different aging periods.

The ResNet-50 backbone adopts a deep residual learning architecture with bottleneck residual blocks and global average pooling. As shown in Table 3, the network maintains a consistent channel expansion pattern and gradually reduces spatial resolution, enabling progressive abstraction of hierarchical visual features. Here, “GAP” and “FC” in Table 3 denote global average pooling and fully connected layers, respectively.

**Table 3:** Network structure.

Layer	Output size	Parameters
Conv1	112×112	7×7,64 stride 2 , 3×3 max pool, stride 2
Conv2_x	56×56	$\left[\begin{array}{c} 1\times 1,128\\ 3\times 3,128\\ 1\times 1,512 \end{array}\right]\times 4$
Conv3_x	28×28	$\left[\begin{array}{c} 1\times 1,64\\ 3\times 3,64\\ 1\times 1,256 \end{array}\right]\times 3$
Conv4_x	14×14	$\left[\begin{array}{c} 1\times 1,256\\ 3\times 3,256\\ 1\times 1,1024 \end{array}\right]\times 6$
Conv5_x	7×7	$\left[\begin{array}{c} 1\times 1,512\\ 3\times 3,512\\ 1\times 1,2048 \end{array}\right]\times 3$
GAP, FC	-	average pool, 1000-d fc, softmax

#### Feature interaction and fusion.

A feature interaction and fusion framework is designed to comprehensively capture the multi-stream visual patterns associated with CRP vintage. Specifically, features are extracted from three parallel ResNet50 branches corresponding to the whole CRP image, the exocarp patch, the albedo patch. These branches are tailored to capture complementary information at different spatial levels: The full-branch, which corresponds to the whole CRP sample, emphasizes global morphology and color distribution; the exocarp-branch focuses on surface texture and pigmentation; and the albedo-branch highlights the internal structure of the albedo.

To enable information exchange while preserving the specialization of each branch, a channel-level interaction mechanism is incorporated into layer2 of the network, which corresponds to the second residual block. This mechanism enables information exchange while preserving the specialization of each branch and adopts an asymmetric two-phase design.

In the forward phase, 10% of the channels from the full-branch feature map *F* are randomly selected and injected into the exocarp-branch *E* and albedo-branch *A*, providing coarse-level contextual cues to enhance local perception. The updated feature maps are defined as $E^{\prime }$ and $A^{\prime }$, and the operation is defined as:

$$E^{\prime }=Replace(E,F,{ℐ}_{10\%}),$$ (7)

$$A^{\prime }=Replace(A,F,{ℐ}_{10\%}),$$ (8)

where ${ℐ}_{10\%}$ denotes the randomly selected 10% of channel indices from the full-branch. The Replace operation substitutes the corresponding channels in the target feature map with those from the full-branch.

In the reverse phase, 5% of the channels from each local branch are sequentially injected back into the full-branch. First, the updated full-branch feature map *F*^(1)^ is obtained by injecting channels from the exocarp-branch:

$$F^{\left(1\right)}=Replace(F,E,{ℐ}_{5\%}).$$ (9)

Then, channels from the albedo-branch are injected to produce the final updated full-branch feature map $F^{\prime }$:

$$F^{\prime }=Replace(F^{\left(1\right)},W,{ℐ}_{5\%}),$$ (10)

where $E^{\prime }$ and $A^{\prime }$ denote the updated exocarp-branch and albedo-branch features after forward injection, *F*^(1)^ is the intermediate full-branch feature map after receiving exocarp-branch information, and $F^{\prime }$ is the final updated full-branch after receiving both local branches.

The injection ratios are selected based on preliminary validation and guided by structural considerations, striking a balance between cross-branch communication and branch-specific specialization. While alternative settings may be explored, this configuration demonstrates stable performance across devices and serves as a reliable default in our framework. Other ratios such as 5% and 15% were also tested during ablation but yielded lower performance, further supporting the current choice.

After interaction, feature fusion is performed. Each updated feature map is first compressed from 2048 to 512 channels using a 1×1 convolution. The three compressed maps are concatenated along the channel dimension to form a fused feature vector $Z\in {\mathbb{R}}^{1536}$. This vector is projected into the category space through a fully connected layer:

$$\hat{y}=Softmax(W_{z}Z+b_{z}),$$ (11)

where *W*_*z*_ and *b*_*z*_ are the weight and bias of the classifier. A cross-entropy loss is applied across all branches to encourage consistent learning and maintain feature alignment:

$$ℒ=-\sum_{k=1}^{C}y_{k}\log({\hat{y}}_{k}),$$ (12)

where *y*_*k*_ is the one-hot encoded ground truth and ${\hat{y}}_{k}$ is the predicted probability for class *k*.

This fusion strategy consolidates multi-stream and region-specific information, enhancing the model’s ability to discriminate between vintage classes while improving robustness across heterogeneous imaging conditions.

#### Meta-learning.

To improve cross-device generalization under heterogeneous imaging conditions, a MAML framework is incorporated. MAML enables the network to learn a device-agnostic initialization that can rapidly adapt to new domains using only a few labeled samples, thus addressing the domain shift problem caused by differences in resolution, color rendering, and sensor characteristics across mobile devices. Each mobile device is regarded as a distinct domain. The iPhone dataset serves as the source domain for meta-training, while Xiaomi and Vivo datasets are used as target domains for meta-testing. During meta-training, four-way five-shot classification tasks are constructed from the source domain using stratified sampling. Each task includes five support images per class and a separate query set for evaluation. The MAML training process involves two optimization loops. In the inner loop, the model performs five steps of gradient descent on the support set to obtain task-specific parameters:

$$\theta_{i}\leftarrow \theta -\alpha \nabla_{\theta }{ℒ}_{support}^{i}(\theta ),$$ (13)

where *θ* is the shared initialization, $\theta_{i}$ is the adapted parameter for task *i*, *α* is the inner-loop learning rate, and ${ℒ}_{support}^{i}$ is the loss over the support set.

In the outer loop, the query losses from multiple tasks are aggregated to update the meta-parameters via:

$$\theta \leftarrow \theta -\beta \nabla_{\theta }\sum_{i}{ℒ}_{query}^{i}(\theta_{i}),$$ (14)

where *β* denotes the meta-learning rate. The meta-update encourages the initialization *θ* to perform well across diverse domains after limited adaptation.

To ensure effective generalization, support and query sets from both target domains are included during meta-testing. The overall meta-training procedure is summarized in Algorithm 2, which outlines the inner-loop adaptation using support samples and the outer-loop meta-update using query losses across tasks. This two-level optimization framework equips the model with the capacity to adapt rapidly to unseen devices under limited supervision.

**Algorithm 2 Meta-training procedure for cross-device CRP classification.**

**Require:** Source domain data ${𝒟}_{src}$ (e.g., iPhone), meta-learning rates *α* (inner), *β* (outer), number of gradient steps *K*

**Ensure:** Meta-initialized model parameters *θ*

1: Initialize model parameters *θ* randomly

2: **for** each meta-training iteration **do**

3:   Sample a batch of tasks $\left\{{𝒯}_{i}\right\}$ from ${𝒟}_{src}$

4:   **for** each task ${𝒯}_{i}$ **do**

5:    Sample support set ${𝒮}_{i}$ and query set ${𝒬}_{i}$

6:    // Inner loop: task-specific adaptation

7:    $\theta_{i}\leftarrow \theta$

8:    **for** *k* = 1 to *K* **do**

9:     $\theta_{i}\leftarrow \theta_{i}-\alpha \nabla_{\theta_{i}}{ℒ}_{{𝒮}_{i}}(\theta_{i})$

10:    **end for**

11:    Compute query loss ${ℒ}_{{𝒬}_{i}}(\theta_{i})$

12:   **end for**

13:   // Outer loop: meta-update across tasks

14:   $\theta \leftarrow \theta -\beta \nabla_{\theta }\sum_{i}{ℒ}_{{𝒬}_{i}}(\theta_{i})$

15: **end for**

16: **return** *θ*

## Results

### Experimental setup and evaluation metrics

In this study, a self-constructed CRP image dataset is used for model training and evaluation. The samples cover multiple vintages, origins, and forgery types, offering high representativeness and discriminability. To assess the generalization capability of the model in cross-device scenarios, all samples were captured using three different consumer-grade mobile devices under natural lighting conditions. Images captured with the iPhone are used for training and validating the base model, while those from Xiaomi and Vivo devices are reserved for subsequent meta-learning-based domain adaptation experiments.

Data partitioning follows a stratified sampling strategy to preserve class distribution across training, validation, and test sets. Specifically, 60% of the data is allocated for training, and 20% each for validation and testing. During training, model parameters are optimized using the Adam optimizer with an initial learning rate of 1e-4, which is dynamically adjusted according to a cosine annealing schedule. Each training cycle spans 80 epochs, with validation accuracy monitored per epoch. Early stopping is applied to prevent overfitting.

To comprehensively evaluate model performance in the CRP classification task, the following four metrics are employed. Accuracy (Acc.): The overall proportion of correctly classified samples; Recall: The model’s ability to identify and cover all categories; F1-score: The harmonic mean of accuracy and recall, particularly useful under class imbalance conditions; Standard deviation of accuracy(STD): Captures the variability in model performance across multiple runs, reflecting its stability and robustness.

### Performance comparison results

To validate the effectiveness of the proposed method, comparative experiments against several representative image classification approaches were conducted. As presented in Table 4, our model achieves the highest Acc. of 95.5%, Recall of 95.6%, and F1-score of 95.5%, outperforming all baselines. It surpasses DenseNet121, the strongest baseline, by 1.2%, 1.3%, and 1.4% on these metrics, respectively. This consistent improvement in Acc., Recall, and F1-score indicates that the gain is not limited to a single indicator, but reflects an overall enhancement of discriminative ability. The multi-input design improves generalization, particularly for fine-grained distinctions, while auxiliary branches help reduce overfitting and enhance robustness. In addition, ten independent runs with different random seeds were conducted to assess reliability. The standard deviation of Acc. is only 1.6%, significantly lower than all other methods, which range from 2.8% to 22.2%. This demonstrates superior consistency and robustness. In summary, the comparative results confirm that the multi-input feature fusion approach significantly improves classification Acc. and robustness in the CRP vintage classification task.

**Table 4:** Performance comparison of different methods on CRP classification.

Method	Acc (%)	Recall (%)	F1-score (%)	STD
VGG16 [22]	92.5	92.4	92.4	**2.8**
GoogleNet [49]	81.5	81.3	79.5	22.2
InceptionV3 [50]	91.8	91.8	91.5	4.8
ResNet50 [23]	85.1	84.8	84.7	9.8
DenseNet121 [24]	**94.3**	**94.3**	**94.1**	**7.4**
RegNet [25]	93.0	92.9	92.8	3.7
2DCNN [51]	86.7	86.7	86.4	5.9
Ours	**95.5**	**95.6**	**95.5**	**1.6**

### Ablation experiment results

To assess the contributions of the feature interaction mechanism and the final fusion module, ablation experiments were conducted using three variants. The baseline model adopts a single-input structure that uses only one regional image, either whole CRP image, exocarp patch, or albedo patch. The no interaction variant employs a multi-input structure where features from the whole, exocarp, and albedo branchs are processed independently and concatenated without cross-channel interaction. The full model integrates a channel-wise interaction module at an intermediate stage and a final feature fusion module prior to classification.

All experiments were conducted under identical training settings and data partitions. Each configuration was run ten times with different random seeds to ensure statistical robustness. Table 5 reports the mean classification Acc. and standard deviation. The results show that the three-branch input structure significantly outperforms single-input baselines, confirming the complementary value of features from the whole CRP image, the exocarp patch, and the albedo patch. The three-branch ResNet50 model achieves 94.4% Acc., 94.3% Recall, and 94.4% F1-score, while the best single-input variant achieves only 85.1%, 84.8%, and 84.7%, respectively. This indicates improvements of 9.3 percentage points in Acc., 9.5 percentage points in Recall, and 9.7 percentage points in F1-score. This gap indicates that single-scale models miss important vintage-related cues, whereas multi-stream inputs provide complementary information for fine-grained recognition.

**Table 5:** Ablation study results comparing different model configurations.

Method	Acc (%)	Recall (%)	F1-score (%)	STD
ResNet50 (whole CRP)	85.1	84.8	84.7	9.8
ResNet50 (exocarp patch)	55.8	56.1	53.1	8.5
ResNet50 (albedo patch)	54.6	54.2	53.0	8.4
Three-branch ResNet50	**94.4**	**94.3**	**94.4**	3.1
Ours	**95.5**	**95.6**	**95.5**	**1.6**

Furthermore, the proposed full model, which incorporates both the feature interaction mechanism and the fusion module, delivers the best overall performance. It achieves an Acc. of 95.5%, a Recall of 95.6%, and an F1-score of 95.5%, while also exhibiting the lowest standard deviation of 1.6% across multiple runs. Compared to the three-branch configuration without interaction and fusion, the proposed full model improves Acc. by 1.1 percentage points, Recall by 1.3 percentage points, and reduces variability from 3.1% to 1.6%. These findings confirm that the integration of feature interaction and fusion enhances the discriminative power and robustness of the model.

**Fig 6:**
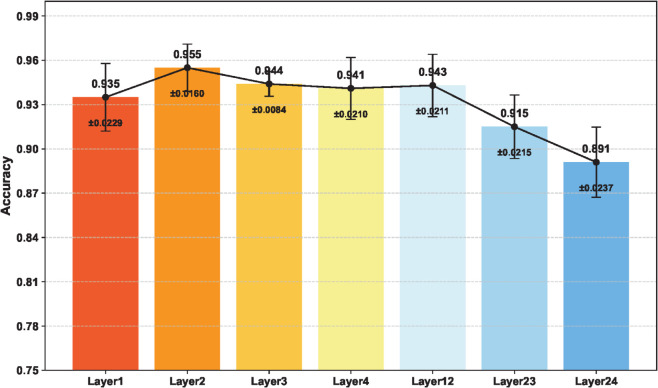
Comparison of classification Acc. across different feature interaction layers.

To assess the impact of interaction layer placement, the feature exchange mechanism was implemented separately at Layer1, Layer2, Layer3, and Layer4 under identical training protocols. Each setting was repeated ten times using different random seeds. The results in Fig 6 show that interaction at Layer2 achieves the highest Acc. of 95.5% with a low standard deviation of 1.6%, indicating its effectiveness in capturing mid-level semantics and fine-grained structural details. In contrast, early-layer interaction at Layer1 offers limited semantic abstraction, resulting in a lower mean Acc. of 93.5% with higher variance. Deeper layers such as Layer3 and Layer4, which involve more abstract features, achieve accuracies of 94.4% and 94.1% respectively, indicating a loss of fine-grained local detail. Applying interaction across multiple layers does not surpass the performance of Layer2, confirming it as the optimal stage for feature fusion. These quantitative results confirm Layer2 as the optimal stage for feature fusion, offering the best trade-off between semantic abstraction and structural fidelity.

### Stability analysis results

**Fig 7:**
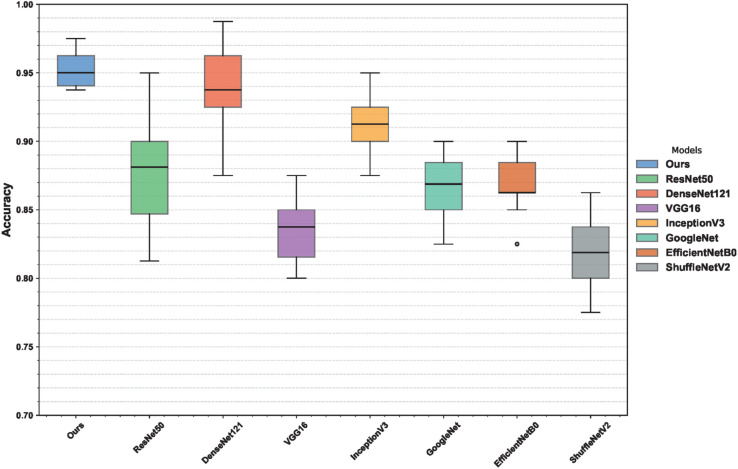
Boxplots illustrating the stability of classification Acc. across multiple independent training runs for different models.

**Fig 8:**
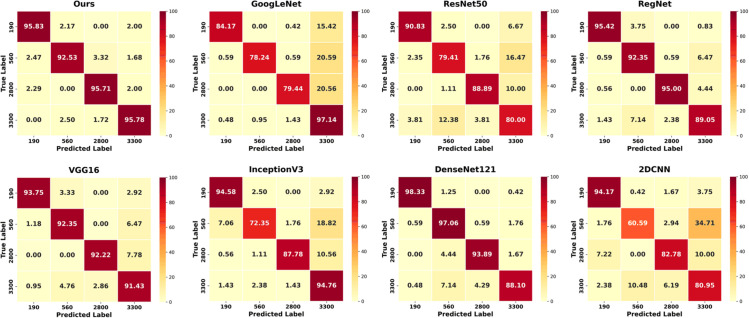
Confusion matrix visualization.

To assess the robustness of the proposed model against variations in initialization and data splits, repeated training experiments were conducted. The results are visualized using boxplots and confusion matrices. Fig 7 shows the distribution of classification Acc. across vintages. Our model achieves a mean Acc. of approximately 0.95 with minimal variance, indicating consistent performance. In contrast, models such as RegNet and DenseNet121, although comparable in mean Acc., exhibit less stability. For example, GoogleNet shows a broad Acc. range from 0.70 to 0.995, and DenseNet121 contains an outlier near 0.75, revealing higher sensitivity to stochastic variation. These findings highlight the superior robustness and generalization of our method. Fig 8 illustrates the model’s classification performance across four vintage categories of CRP. Blacker shades indicate higher prediction Acc., while lighter ones reflect misclassification trends. Our method achieves 95.83%, 92.53%, 95.71%, and 95.78% Acc. for the 190, 560, 2800, and 3300 categories respectively, with misclassification rates remaining below 3.5%. In contrast, other models perform less reliably. For example, GoogleNet reaches only 78.24% on the 560 category, misclassifying 20.59% as 3300, while 2DCNN drops to 60.59%, with 34.73% wrongly predicted as 190. Even RegNet, though competitive, is consistently outperformed. These results confirm the superior Acc., robustness, and cross-category reliability of our method.

### Cross-domain evaluation via meta-learning

Two evaluation strategies are compared to assess the model’s ability to generalize across different mobile devices. In the first strategy, the model is trained on the iPhone dataset and directly applied to images captured by other devices without any further adaptation. This direct transfer setting reflects the performance drop typically caused by device-induced distribution shifts. The second strategy employs the proposed meta-learning framework. The model is meta-trained using data from the iPhone and then adapted to each target domain, namely Xiaomi and Vivo, using a small support set. Specifically, a four-way five-shot configuration is used: five labeled images per class are selected for adaptation, while the remaining images serve as the query set. This setup reflects realistic deployment conditions where only limited labeled data are available when encountering new smartphone cameras.

**Fig 9:**
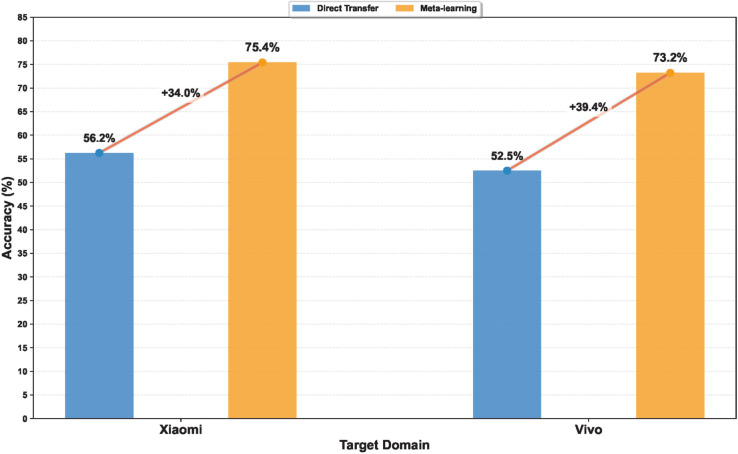
Comparative results of cross-domain classification Acc. between direct transfer and meta-learning adaptation on Xiaomi and Vivo target domains.

The classification results are summarized in Fig 9. On the Xiaomi dataset, Acc. improves from 56.2% under direct transfer to 75.4% with meta-learning,representing a relative improvement of 34.2%. On the Vivo dataset, Acc. increases from 52.5% to 73.2%, corresponding to a 39.4% relative improvement. All results are averaged over ten independent runs using randomly sampled support-query splits to ensure statistical robustness. These findings confirm that the proposed meta-learning approach substantially enhances cross-device adaptability. By learning a domain-agnostic initialization, the model can rapidly adjust to device-specific imaging variations with minimal supervision. This capability supports consistent classification performance across diverse mobile imaging conditions without the need for extensive retraining or manual relabeling. Overall, these results show that device differences cause a large performance drop under direct transfer, and that the proposed meta-learning effectively reduces this gap.

### Visualization experiments results

**Fig 10:**
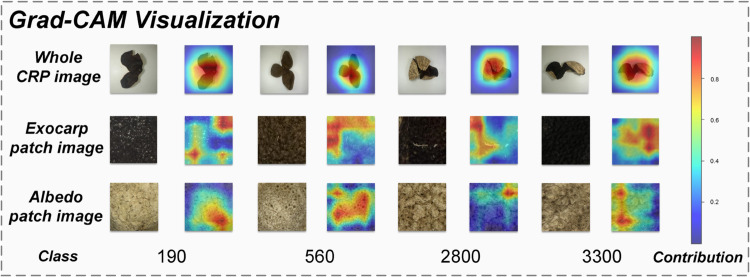
Grad-CAM visualization.

To analyze the regions of CRP that the model focuses on during classification, Gradient-weighted Class Activation Mapping (Grad-CAM) [52] was employed to visualize the model’s prediction process. Grad-CAM facilitates the interpretation of critical features by generating heatmaps that highlight the key areas within the input images influencing the model’s decisions. Fig 10 presents the Grad-CAM visualization results. Each pair of columns corresponds to samples at different price points and their associated Grad-CAM heatmaps, while each row represents one of the three input types: the whole CRP image, the exocarp patch, the albedo patch. The color intensity indicates the relative importance of different regions, with warmer colors such as red denoting higher relevance and cooler colors such as blue indicating lower influence. The results show that the model primarily focuses on the whole CRP image to make predictions, while the exocarp and albedo patches provide complementary cues related to texture and brightness. These findings confirm that the whole CRP image serves as the dominant input for classification, and that the additional regional images enhance discriminative capacity by supplying fine-grained structural and visual details.

**Fig 11:**
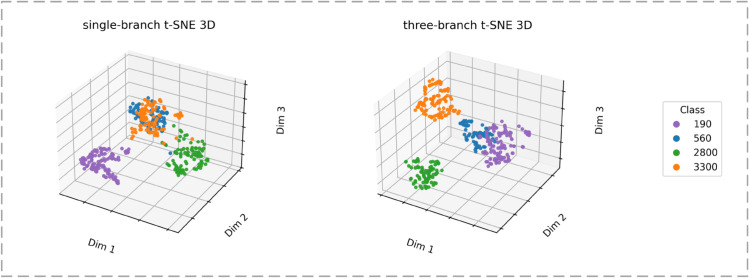
3D t-SNE visualization.

To analyze the separability of the features learned by different network structures, 3D three-dimensional t-distributed stochastic neighbor embedding (t-SNE) [53] was employed to visualize the high-dimensional embeddings of the single-stream model and the proposed three-stream network. t-SNE projects the extracted features into a three-dimensional space by preserving local neighborhood relationships among samples. Fig 11 presents the visualization results. The results show that the single-stream model exhibits initial class grouping but still suffers from evident inter-class overlap, particularly between confusing categories such as 560 and 3300. In contrast, the three-stream network produces more compact intra-class distributions and clearer inter-class boundaries, indicating that multi-stream learning enhances discriminative representation by capturing fine-grained details.

## Discussion

The proposed method achieved 95.5% accuracy in classifying CRP vintages on iPhone images, with over a 34% relative improvement in cross-device generalization compared to direct transfer learning. This validates our hypothesis that combining multi-stream feature extraction with meta-learning effectively mitigates domain shift across different imaging devices. The model captures visual differences in the exocarp, albedo, and whole morphology of CRP. Channel-level feature interaction further enhances feature discrimination, while the meta-learning component improves adaptability to device differences. These advantages make the method a practical reference for onsite vintage classification, quality inspection, pricing, and authenticity verification in the CRP market. This is particularly important in wholesale markets and retail stores, where vintage and authenticity directly affect product value.

Nevertheless, the study has certain limitations. The dataset includes only four vintage categories, and the imaging devices used are limited in type and diversity, which may affect the model’s generalization to broader market scenarios. Additionally, the current model exhibits performance degradation under challenging conditions such as glare, occlusion, and inconsistent lighting. These factors constrain its robustness and practical deployment.

Future work will address these limitations by expanding the dataset to include more vintages and devices, as well as images captured under diverse environmental conditions. In our future work, the illumination normalization and enhancement algorithms will be employed to address lighting variations in images. Leveraging existing methods such as Multi-Scale Retinex, Zero-DCE, RetinexNet and EnlightenGAN to improve color constancy and enhance images captured under non-uniform or low-light conditions. We also plan to incorporate spectral and fine-grained textural features to strengthen feature representation. Furthermore, strategies such as multimodal fusion, self-supervised learning, and incremental learning will be explored to enhance the model’s adaptability and scalability, ultimately supporting real-world applications in CRP market inspection.

## Conclusions

This study presents a multi-stream feature fusion and meta-learning framework for vintage classification of CRP images captured using mobile devices. Key regions including the exocarp and the albedo patches are localized through object detection and bounding box refinement, enabling region-specific feature extraction. A three-branch network with intermediate feature interaction supports multi-stream representation learning. To address domain shift across devices, a MAML-based meta-learning module improves adaptation and generalization. Experiments on a four-class dataset show that the proposed method outperforms baseline models in Acc., F1-score, and robustness. Cross-device results confirm its effectiveness under limited data conditions.

These findings demonstrate the potential of combining deep learning with mobile imaging for practical CRP classification. The method provides a non-destructive, efficient, and scalable solution, offering valuable technical support for quality inspection, pricing, and authenticity verification in the CRP market.
